# Enhancement of accuracy and efficiency for RNA secondary structure prediction by sequence segmentation and MapReduce

**DOI:** 10.1186/1472-6807-13-S1-S3

**Published:** 2013-11-08

**Authors:** Boyu Zhang, Daniel T Yehdego, Kyle L Johnson, Ming-Ying Leung, Michela Taufer

**Affiliations:** 1 Department of Computer and Information Sciences, University of Delaware, Newark, DE 19716, USA; 2 Computational Science Program, The University of Texas at El Paso, El Paso, TX 79968, USA; 3 Bioinformatics Program, The University of Texas at El Paso, El Paso, TX 79968, USA; 4 Department of Biological Sciences, The University of Texas at El Paso, El Paso, TX 79968, USA; 5 Department of Mathematical Sciences, The University of Texas at El Paso, El Paso, TX 79968, USA

## Abstract

**Background:**

Ribonucleic acid (RNA) molecules play important roles in many biological processes including gene expression and regulation. Their secondary structures are crucial for the RNA functionality, and the prediction of the secondary structures is widely studied. Our previous research shows that cutting long sequences into shorter chunks, predicting secondary structures of the chunks independently using thermodynamic methods, and reconstructing the entire secondary structure from the predicted chunk structures can yield better accuracy than predicting the secondary structure using the RNA sequence as a whole. The chunking, prediction, and reconstruction processes can use different methods and parameters, some of which produce more accurate predictions than others. In this paper, we study the prediction accuracy and efficiency of three different chunking methods using seven popular secondary structure prediction programs that apply to two datasets of RNA with known secondary structures, which include both pseudoknotted and non-pseudoknotted sequences, as well as a family of viral genome RNAs whose structures have not been predicted before. Our modularized MapReduce framework based on Hadoop allows us to study the problem in a parallel and robust environment.

**Results:**

On average, the maximum accuracy retention values are larger than one for our chunking methods and the seven prediction programs over 50 non-pseudoknotted sequences, meaning that the secondary structure predicted using chunking is more similar to the real structure than the secondary structure predicted by using the whole sequence. We observe similar results for the 23 pseudoknotted sequences, except for the NUPACK program using the centered chunking method. The performance analysis for 14 long RNA sequences from the *Nodaviridae *virus family outlines how the coarse-grained mapping of chunking and predictions in the MapReduce framework exhibits shorter turnaround times for short RNA sequences. However, as the lengths of the RNA sequences increase, the fine-grained mapping can surpass the coarse-grained mapping in performance.

**Conclusions:**

By using our MapReduce framework together with statistical analysis on the accuracy retention results, we observe how the inversion-based chunking methods can outperform predictions using the whole sequence. Our chunk-based approach also enables us to predict secondary structures for very long RNA sequences, which is not feasible with traditional methods alone.

## Background

### RNA molecules

Ribonucleic acid (RNA) is made up of four types of nucleotide bases: adenine (A), cytosine (C), guanine (G), and uracil (U). A sequence of these bases is strung together to form a single-stranded RNA molecule. RNA plays important roles in many biological processes including gene expression and regulation. RNA molecules vary greatly in size, ranging from nineteen nucleotide bases in microRNAs [1] to long polymers of over 30,000 bases in complete viral genomes [2]. Although an RNA molecule is a linear polymer, it tends to fold back on itself to form a 3-dimensional (3D) functional structure, mostly by pairing complementary bases. Among the four nucleotide bases, C and G form complementary base pairs by hydrogen bonding, as do A and U; in RNA (but not DNA), G can also base pair with U residues. The overall stability of an RNA structure element is determined by the "minimal free energy" defined as the amount of energy it would take to completely unpair all of the base pairs that hold it together (e.g., by denaturing it with heat).

**Figure 1:**
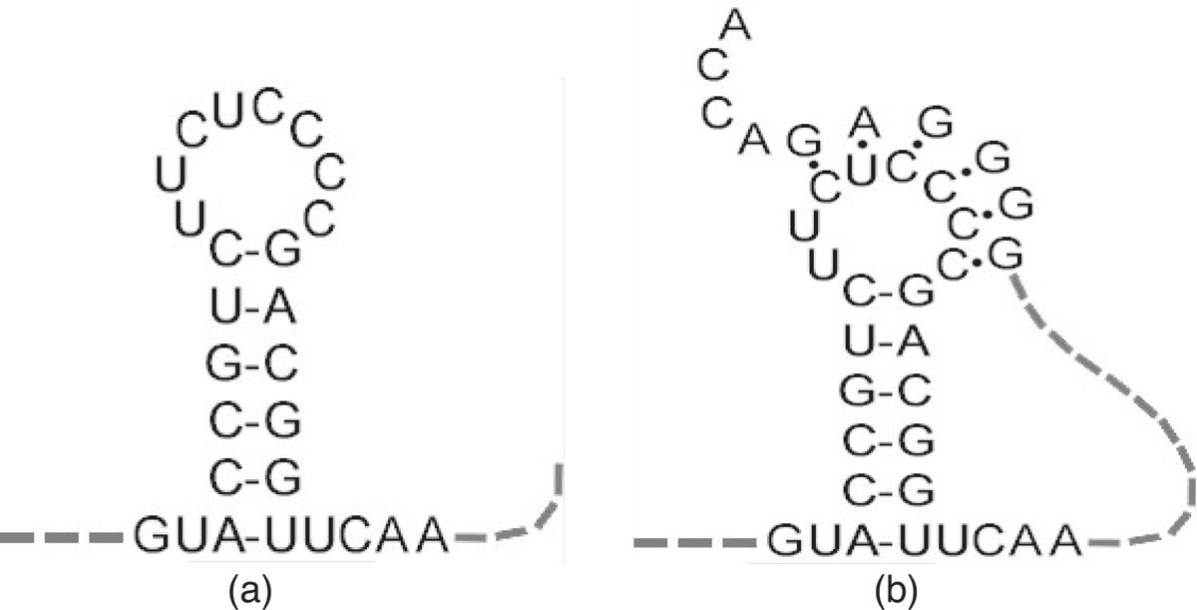
**Basic elements in RNA secondary structures**. The stem loop (a) and pseudoknot (b).

**Figure 2:**
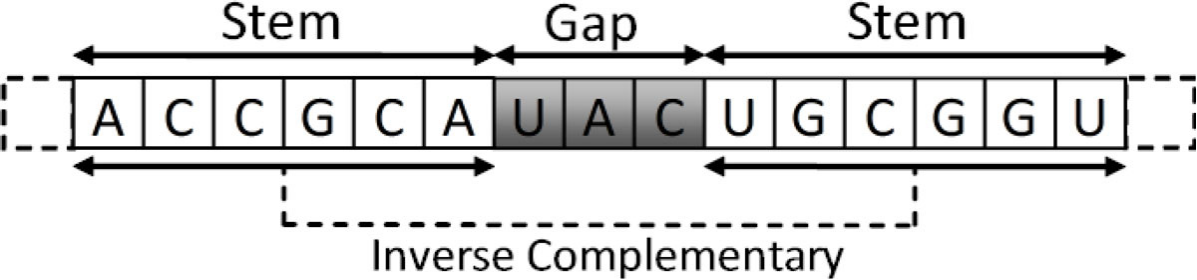
**Example of an inversion**. An inversion with stem length 6 and gap size 3.

The 3D structure of an RNA molecule is often the key to its function. Because of the instability of RNA molecules, experimental determination of their precise 3D structures is a time-consuming and rather costly process. However, useful information about the molecule can be gained from knowing its secondary structure, i.e., the collection of hydrogen-bonded base pairs in the molecule [3]. RNA secondary elements can be classified into two basic categories: stem-loops and pseudoknots (see Figure 1). Both kinds of secondary structure elements, which have been implicated in important biological processes like gene expression and gene regulation [4], must contain at least one inversion, i.e., a string of nucleotides followed closely by its inverse complementary sequence. Figure 2 shows an example of an inversion, with the 6-nucleotide string "ACCGCA" followed by its inverse complementary sequence "UGCGGU" after a gap of three nucleotides.

### RNA secondary structure predictions

Secondary structures are crucial for the RNA functionality and therefore the prediction of the secondary structures is widely studied. Development of mathematical models and computational prediction algorithms for stem-loop structures began in the early 1980's [5-7]. Pseudoknots, because of the extra base-pairings involved, must be represented by more complex models and data structures that require large amounts of memory and computing time to obtain the optimal and suboptimal structures with minimal free energies. As a result, development of pseudoknot prediction algorithms began in the 1990's [8,9].

Most existing secondary structure prediction algorithms are based on the minimization of a free energy (MFE) function and the search for the most thermodynamically stable structure for the whole RNA sequence. Searching for a structure with global minimal free energy may be memory and time intensive, especially for long sequences with pseudoknots. To overcome the tremendous demand on computing resources, various alternative algorithms have been proposed that restrict the types of pseudoknots for possible prediction in order to keep computation time and storage size under control. Yet, most programs available to date for pseudoknot structure prediction can only process sequences of limited lengths on the order of several hundred nucleotides. These programs, therefore, cannot be applied directly to larger RNA molecules such as the genomic RNA in viruses, which may be thousands of bases in length. At the same time, minimal energy configurations may not be the most favorable structures for carrying out the biological functions of RNA, which often require the RNA to react and bind with other molecules (e.g., RNA binding proteins). Our current work suggests that local structures formed by pairings among nucleotides in close proximity and based on local minimal free energies rather than the global minimal free energy, may better correlate with the real molecular structure of long RNA sequences. This hypothesis has yet to be supported by more detailed experimental evidence. If proven correct, our approach will open the door to a new generation of programs based on segmenting long RNA sequences into shorter chunks, predicting the secondary structures of each chunk individually, and then assembling the prediction results to give the structure of the original sequence.

In our previous work, we had proposed to predict secondary structures for long RNA sequences using three steps: (1) cut the long sequence into shorter, fixed-size chunks; (2) predict the secondary structures of the chunks individually by distributing them to different processors on a Condor grid; and (3) assemble the prediction results to give the structure of the original sequence [10]. We used this approach on the genome sequences of the virus family *Nodaviridae*, leading to the discovery of secondary structures essential for RNA replication of the *Nodamura *virus [11]. However, the study also identified the necessity of having a more effective segmentation strategy for cutting the sequence so that the predicted results of the chunks can be assembled to generate a reasonably accurate structure for the original molecule. Indeed, the selection of cutting points in the original RNA sequence is a crucial component of the segmenting step. In this paper, we propose to approach the problem by identifying inversion excursions in the RNA sequence and cutting around them. We consider two alternative inversion-based segmentation strategies: the centered and optimized chunking methods. Both methods identify regions in the sequence with high concentrations of inversions and avoid cutting into these regions. In the centered method, the longest spanning inversion clusters are centered in the chunks, while in the optimized method, the number of bases covered by inversions is maximized. Preliminary results have been presented in the authors' work [12,13].

### MapReduce and Hadoop

The prediction of RNA secondary structures for long RNA sequences based on sequence segmentation can be performed in parallel, thus benefiting from parallel computing systems and paradigms. We use the well-known MapReduce framework Hadoop for our parallel predictions. The MapReduce paradigm is a parallel programming model that facilitates the processing of large distributed datasets, and it was originally proposed by Google to index and annotate data on the Internet [14]. In this paradigm, the programmer specifies two functions: map and reduce. The map function takes as input a key *k*_1 _and value *v*_2 _pair, performs the map function, and outputs a list of intermediate key and value pairs which may be different from the input *list *〈*k*_2_*, v*_2_〈 - i.e., *Map *〈*k*_1_*, v*_1_〉 *→ list *〈*k*_2_*, v*_2_〉. The runtime system automatically groups all the values associated with the same key and forms the input to the reduce function. The reduce function takes as input a key and values pair 〈*k*_2_*, list*(*v*_2_)〉, performs the reduce function, and outputs a list of values - i.e., *Reduce *〈*k*_2_*, list*(*v*_2_)〉 *→ list *〈*v*_3_〉. Note that the input values to reduce is the list of all the values associated with the same key.

MapReduce is appealing to scientific problems, including the one addressed in this paper, because of the simplicity of programming, the automatic load balancing and failure recovery, as well as the scalability. It has been widely adapted for many bioinformatics applications. For example, Hong et al. designed an RNA-Seq analysis tool for the estimation of gene expression levels and genomic variant calling [15], and Langmead et al. designed a next-generation sequencing tool based on MapReduce Hadoop [16]. To the best of our knowledge, our work is the first one to adapt MapReduce into secondary structure predictions of long

RNA sequences. Preliminary work on the reasoning behind adapting RNA secondary structure predictions to the MapReduce paradigm can be found at [17].

## Method

### Workflow for parallel chunk-based predictions

Rather than predicting the RNA sequence as a whole, we cut each sequence into chunks and predict each chunk independently before merging the predictions into the whole secondary structure. As the cutting process can be performed in different ways, the search for effective ways to cut sequences can require a large search space and generate a large number of independent prediction jobs that can potentially be performed in parallel. The workflow for a parallel chunk-based RNA secondary structure prediction and accuracy assessment consists of the following four steps: (1) *chunking*: each RNA sequence is cut into multiple chunks (or segments) according to various chunking algorithms and parameters; (2) *prediction*: the secondary structure for each chunk is predicted independently by using one or more prediction programs; (3) *reconstruction*: the whole secondary structure of a sequence is reconstructed from predicted structures, one for each chunk; and (4) *analysis*: reconstructed structures are compared against known structures to assess prediction accuracies.

**Figure 3:**
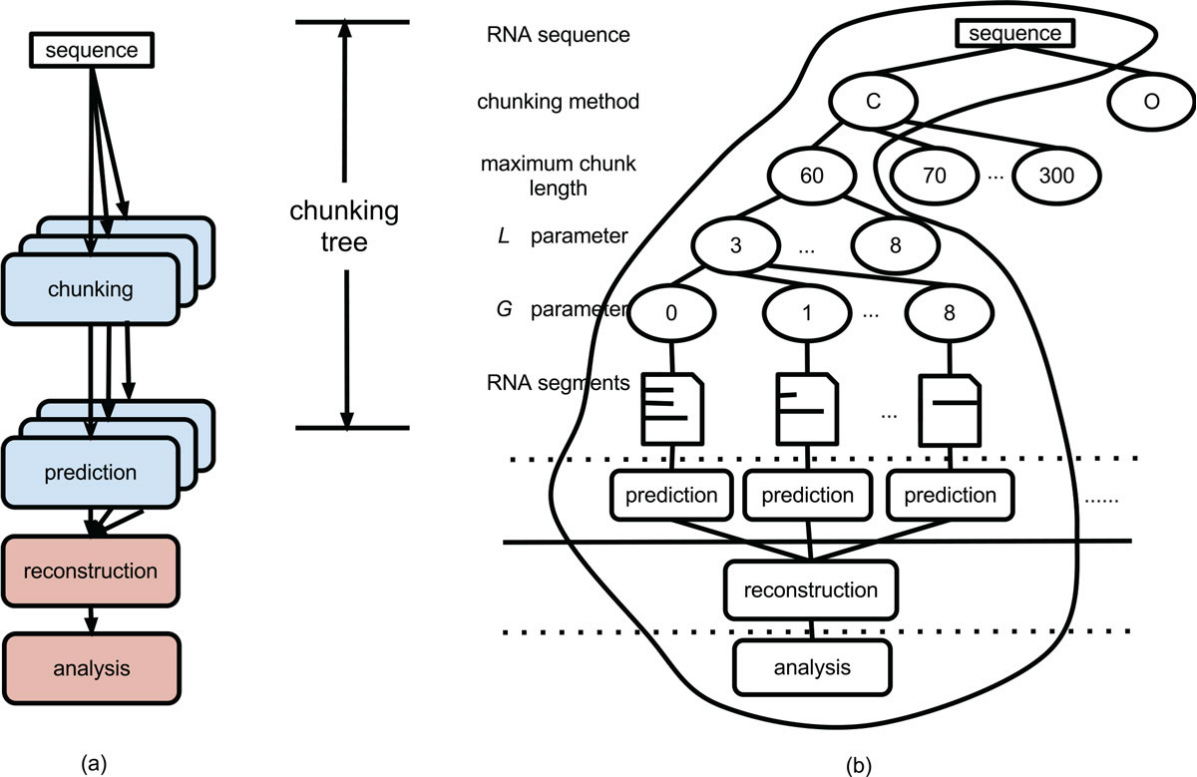
**Workflow of chunk-based RNA predictions**. Workflow of the chunk-based RNA secondary structure prediction framework (a) and example of searching path (b).

Figure 3.a shows the prediction workflow. Note that the chunks do not necessarily have the same length: the lengths depend on the chunking method and parameters used. Also note that the chunk's prediction time and memory usage can vary based on the number of nucleotides in the chunk and the prediction program used. In most prediction programs the time and memory used do not grow linearly but exponentially with the number of nucleotides, with the exponential factor depending on the program complexity and its capability to capture complex RNA secondary structures such as pseudoknots.

### Chunking process based on inversions

Given a long RNA sequence, we identify regions with high concentrations of inversions by using an adapted version of the "Palindrome" program in the EMBOSS package [18], which is a free open source software analysis package. Two main reasons for adapting the EMBOSS Palindrome program are as follows: the original program works correctly on DNA but not RNA sequences and does not support G-U pairing that we plan to include in our adaptation. Our adapted program, InversFinder, is written in Java and is available for download at http://rnavlab.utep.edu. InversFinder requires a text file containing the RNA sequence in FASTA format as input. The minimum stem length *L *and maximum gap size *G *of the inversion are parameters specified by the user.

The chunking step relies on a general excursion approach first formulated in [19], which has already been applied to a variety of sequence analysis problems but not to RNA secondary structure predictions. In many bioinformatics applications, the problem calls for identifying high concentration regions of a certain property in the nucleotide bases of biomolecular sequences. For example, replication origins in viral genomes have been predicted by looking for regions that are unusually rich in the nucleotides A and T in DNA sequences [20]. In this paper, we follow the same approach for RNA sequences, but our focus is whether or not the nucleotide base is found inside an inversion. We refer to the excursions generated by this property as "inversion excursions." The excursion method requires assigning a positive score to each nucleotide if it is a part of an inversion (including the two stems and the gap between them), and a negative score if it does not. We go through the entire nucleotide sequence accumulating the scores to form inversion excursions.

**Figure 4:**
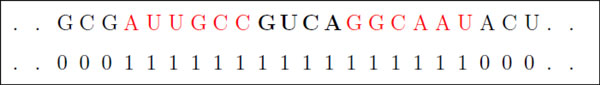
**Binary sequence around an inversion**. The binary sequence around an inversion. If a nucleotide base is included in an inversion identified by the InversFinder program, it is given a value of "1"; if not, it is assigned a value of "0".

To facilitate the analysis, we use a parsing program to convert an RNA sequence into a binary sequence with the same length. If a nucleotide base is included in an inversion identified by the InversFinder program, it is given a value of "1"; if not, it is assigned a value of "0," as illustrated in Figure 4. Each "1" in the binary sequence is given a score of 1, and each "0" a negative score of *s *which is determined as follows: we consider the binary sequence as a realization of a sequence of independent and identically distributed (i.i.d.) random variables, *X*_1_*, X*_2_*,..., X_n_*, where *n *is the length of the RNA sequence (i.e., number of bases). These random variables take values of either 1 or *s*. Let *p *= *Pr*(*X_i _*= 1) and *q *= 1 *- p *= *Pr*(*X_i _*= *s*). The parameter *p *is naturally estimated by the percentage of bases contained in one or more inversions in the RNA sequence, i.e., the percentage of "1"s in the binary sequence. We require that the expected score per base *μ*= *p *+ q * s to be negative. This requirement prevents the tendency of favoring long segments to be high scoring segments. As done in [20] and other applications, the value of *s *can be conveniently selected by giving *μ*a value of *-*0.5 and then determining the value of *s *according to Equation 1.

(1) $$s=\left\lfloor \frac{\mu -p}{q}\right\rfloor$$

The excursion score *E_i _*at Position *i *of the sequence is defined recursively as in Equations 2 and 3.

(2) $$E_{0}=0$$

(3) $$E_{i}=max\left(E_{i-1}+X_{i},0\right)\;for\;1\leq i\leq n$$

**Figure 5:**
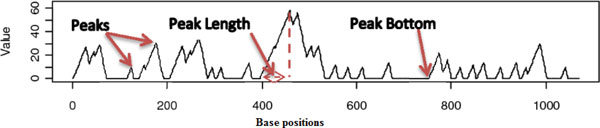
**Peaks, peak bottoms, and peak lengths**. An excursion plot with peaks, peak bottoms, and peak lengths. Rising stretches in the plot indicate the presence of inversions.

An excursion starts at a point *i *where *E_i _*is zero, continues with a number of rising and falling stretches of positive values, and ends at *j > i *where *j *is the next position with *E_j _*= 0. The score then stays at zero until it becomes positive again when the next excursion begins. Plotting the excursion scores along the nucleotide positions of the RNA sequence offers an effective visualization of how inversion concentrations vary along the sequence. This plot can serve as a guide for choosing the cutting points for the segmentation process. Figure 5 shows an example of an excursion plot. Note that rising stretches in the plot indicate the presence of inversions.

After generating the excursion plot, we identify the positions, called peaks, where the excursion scores are local maxima. Then, the bottom of each peak, which is the last position with a zero excursion score right before the peak, is located. After that, the length of the peak (the location difference between a peak and its peak bottom) is calculated. Note that since we require chunk lengths to be smaller than a prescribed maximum *c*, peak lengths greater than *c *have to be flagged and analyzed separately. Figure 5 also shows examples of peaks, peak bottoms, and peak lengths. Peaks are sorted in decreasing order based on their excursion scores. The sorted peaks are then used to cut sequences in chunks by the centered and optimized chunking methods.

#### Centered chunking method

The centered method cuts the sequence by identifying inversions and building the chunks around them. The objective is to segment the RNA sequence in such a way as to avoid losing structural information as much as possible by centering the longest spanning inversion clusters in the chunks. After peaks are identified, they are sorted in decreasing order of their excursion values. The peak with the highest excursion value is considered first, then the second highest peak is considered, and so on. The algorithm stops either when all the peaks are exhausted or when all the inversion regions of the sequence (i.e., all "1"s in the binary sequence) have been included in the chunks, whichever occurs first. Overlapping chunks are adjusted so that any nucleotide base is captured by only one chunk, with priority given to the peak with a higher excursion score.

**Figure 6:**
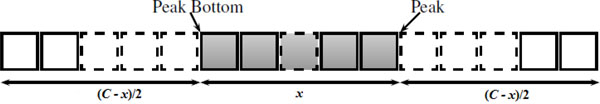
**Centered chunking method**. Centered chunking method where *x *= peak length. We take the sequence segment between the peak bottom and the position of the very last peak in the excursion, and place the sequence segment in the center of the chunk.

For each of the selected peaks, the positions of the inversions or peak length positions are centered within the maximum chunk-length of *c *bases where *c *is defined by the user. We start at the bottom of this peak and follow the excursion until it returns to 0 the very next time and locate the position of the very last peak before the excursion returns to 0. We take the sequence segment between the peak bottom and the position of the very last peak and place the sequence segment in the center of the chunk as illustrated in Figure 6. Suppose this centered segment contains *x *nucleotide bases. If (*c - x*) is even, then the resulting chunk will have (*c - x*)*/*2 bases on each side of the centered segment. If (*c - x*) is odd, then we will adjust the lengths on each side to the integers below and above (*c - x*)*/*2, allowing one side (chosen at random) to have one more nucleotide base than the other.

**Figure 7:**
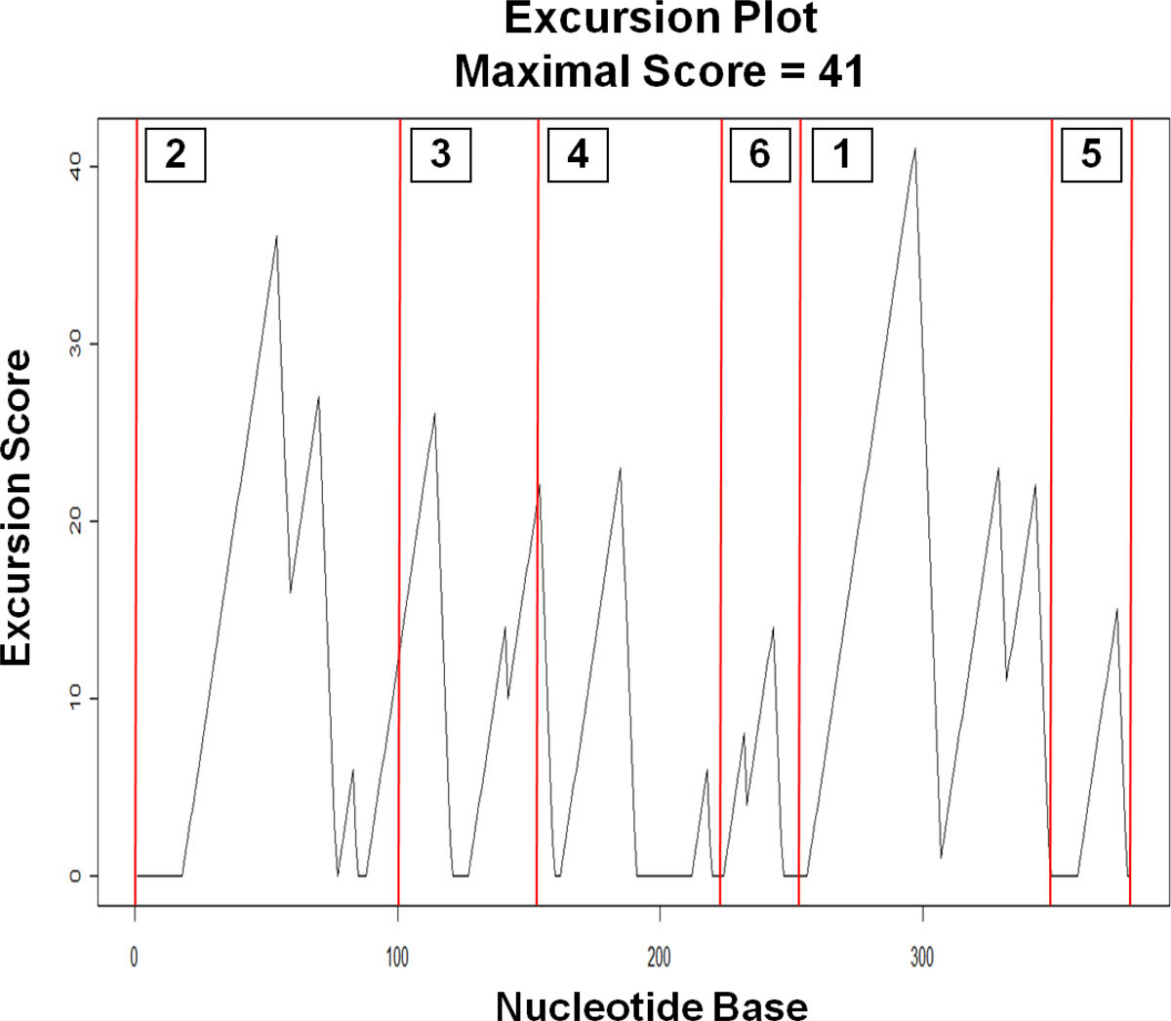
**Example of chunking with centered method**. Six chunks are obtained using the centered method for the 379-base RNA sequence RF00209_A in the RFAM database.

As an example, we applied the aforementioned method to an RNA sequence, that is, the 379-base RNA sequence RF00209_A in the RFAM database [21]. As shown in Figure 7, the sequence is segmented into six chunks using the centered chunking method. These six segments cover the entire sequence. Labels 1 through 6 in Figure 7 represent the six segments with decreasing order of peak excursion scores. After the peak scores are sorted, the peak with the highest excursion score is considered first. In this example, we use the maximum chunk-length *c *= 100. The highest peak is found at Position 297 with peak bottom at 257. As there are other inversions after the highest scoring peak, we follow the entire excursion to the end at Position 356. After locating the last peak in this excursion at 343, we center the sequence segment from 257 to 343 to produce the chunk covering the 100 positions from 250 to 349, then the second highest scoring peak at Position 54 is considered and the above procedure is repeated. This time, the peak bottom is at Position 19 and the last peak before the end of this excursion is at Position 70. Centering the segment consisting of Positions 19 *- *70 in a chunk of 100 would require 24 positions on each side, extending the chunk beyond the beginning of the sequence; we therefore adjust the chunk to start at Position 1 instead. Note that during the segmentation process, we might get a chunk that overlaps with previously established chunks. In those cases, we have to reconcile the situation by reducing one of the chunk lengths. For example, after establishing the first two chunks (labels 1 and 2 in Figure 7), the next highest peak to be processed is at Position 114, with peak bottom at Position 89. Centering this peak produces a chunk from Positions 52 to 151, overlapping with Chunk 2. We resolve such conflicts by giving priority to the chunk with the higher number of bases within completely contained inversions. With this rule, we give priority to Chunk 2, and reduce Chunk 3 to Positions 101 - 151. The process continues for the remaining Chunks 4 - 6.

#### Optimized chunking method

**Figure 8:**
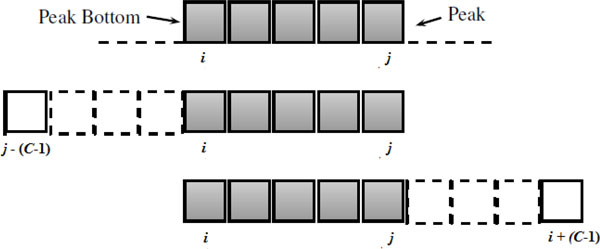
**Optimized chunking method**. Chunks by optimized method with peak spanning Positions *i*-*j*. All segments with length *c *between Positions *j - *(*c - *1) and *i *+ (*c - *1) are considered. The chunk with the highest inversion score is selected.

In the optimized method, cutting points are decided by choosing a segment containing the peak in an optimal position that yields the highest inversion scores for the segment. The score is defined as the total number of nucleotide bases contained in the inversions that are entirely within the chunk. For example, consider a peak with peak length spanning the nucleotide bases between *i *and *j *and then all the chunks of size *c *covering this peak, that is, all segments with length *c *between Positions *j - *(*c - *1) and *i *+ (*c - *1) are considered (see Figure 8). The chunk with the maximum inversion score is then selected. Beginning with the highest peak, the above process is repeated until either all the peaks are utilized or all the inversions of the sequence are contained in established chunks, whichever occurs first. When chunks overlap, the cutting points are adjusted in a similar way as described for the centered method. The optimized method ensures that peak length positions are included within a chunk but not necessarily in the center of the chunk.

**Figure 9:**
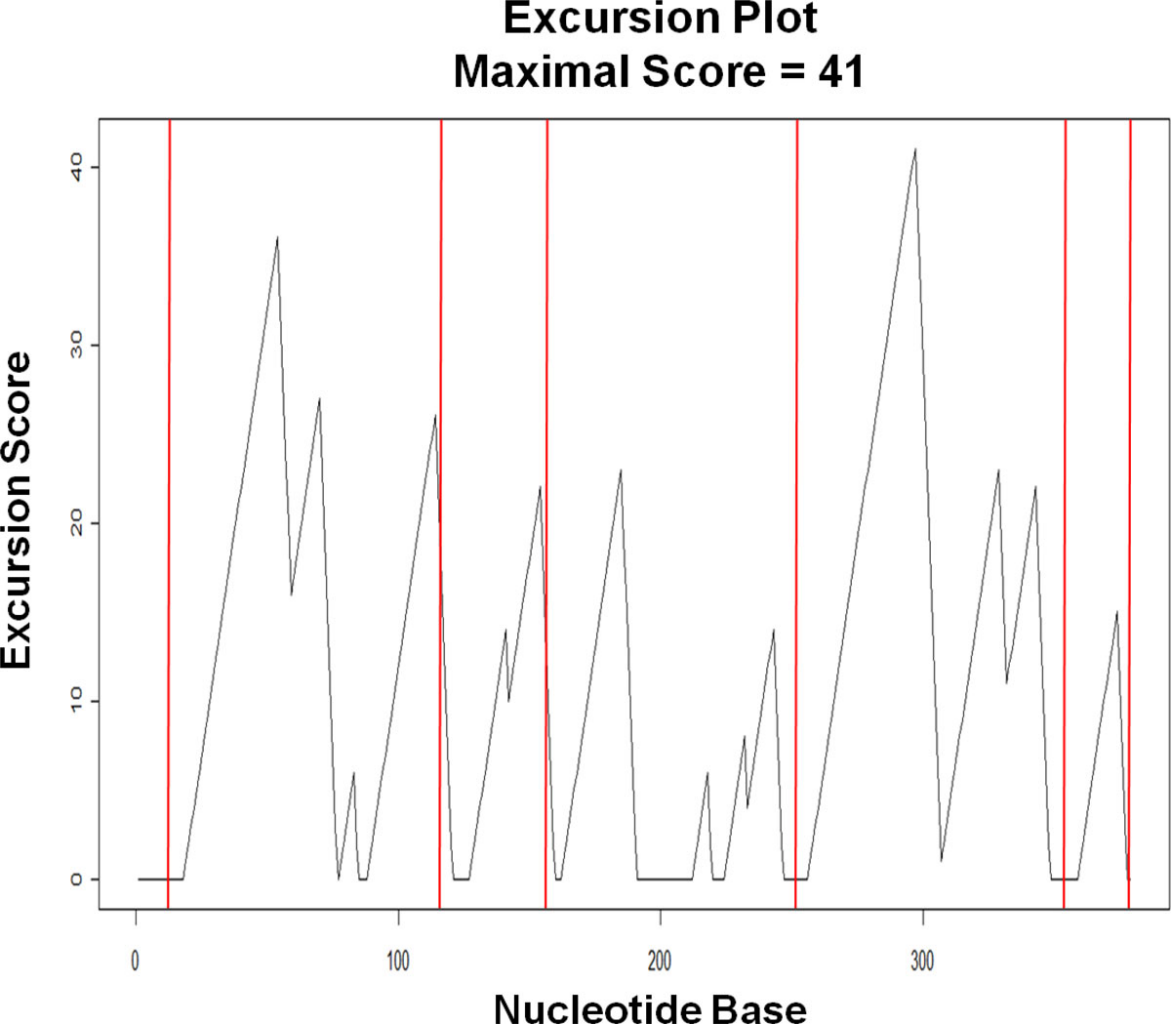
**Example of chunking from optimized method**. Five chunks are obtained using the optimized method for the 379-base RNA sequence RF00209_A in the RFAM database. The chunks covering all except the first 18 positions of the sequence.

As an example, we applied the optimized method to the same RF00209_A RNA sequence file from the RFAM database, as shown in Figure 9. The optimized method produced only 5 chunks covering all except the first 18 positions of the sequence. It can be seen from Figure 9 that this method avoids cutting into those sequence segments with rising excursion scores preceding the peaks. Also, the chunks produced by the optimized method cover only 96.3% of the sequence, leaving out those parts of the sequence where no inversions are found; therefore, the wasting of computing resources is minimal in the optimized method.

#### Regular chunking method

The regular chunking method is the simplest method of segmentation and is used as a reference method in this paper. This method cuts the nucleotide sequence regularly into chunks of a specified maximum chunk-length *c *until the sequence is exhausted.

**Figure 10:**
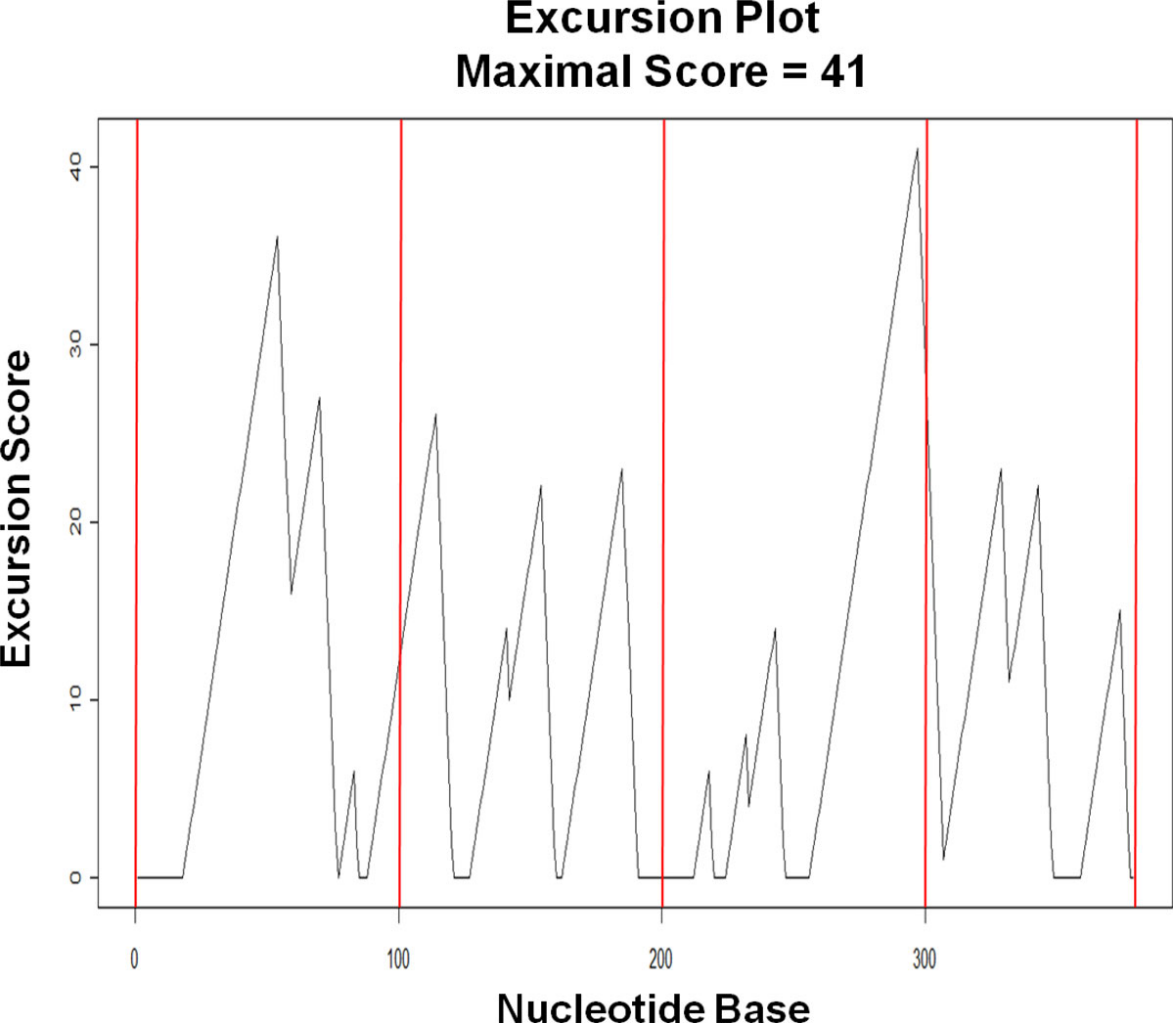
**Example of chunking from regular method**. With *c *= 100, the sequence RF00209_A is cut into four chunks positioned at 1 *- *100, 101 *- *200, 201 *- *300, and 301 *- *379.

For example, with *c *= 100, the sequence RF00209_A from the RFAM database with 379 bases will be cut into four chunks made up of nucleotide Positions 1 *- *100, 101 *- *200, 201 *- *300, and 301 *- *379 (Figure 10). Obviously, rising stretches in an excursion plot, which indicate the presence of inversions and are likely to be part of secondary structures, can often be cut by this method. As a result, it is relatively easy to lose important structural information. Intuitively, one expects that both the centered and optimized methods, which take the inversion locations into account when placing the chunks, perform better in retaining the secondary structure information in the sequences.

### Prediction based on well-known algorithms

After the RNA sequence is cut into chunks, the structure of each chunk is predicted independently using well-known algorithms and their programs. We use the same prediction algorithms to predict the entire sequence without chunking. We employ seven commonly used prediction programs to test the chunking methods. The programs that predict structures only for non-pseudoknotted sequences are UNAFOLD (2008) and RNAfold (1994). The programs that predict both pseudoknotted and non-pseudoknotted sequences are IPknot (2011), pknotsRG (2007), HotKnots (2005), NUPACK (2004), and PKNOTS(1998). These prediction programs, which typically involve some form of minimization of free energy, maximization of expected accuracy, or dynamic programming models in their algorithms, are all publicly available.

### Reconstruction based on concatenation

The results of the chunk predictions are assembled to build a whole secondary structure. Currently, our framework simply concatenates all these predicted secondary structures to give the secondary structure for the whole sequence. This is possible because the cutting does not allow any overlap between two consecutive chunks. More sophisticated reconstruction methods that include partial chunk overlaps can be used with minor changes to our framework.

### Accuracy analysis based on comparisons with known structures

**Figure 11:**
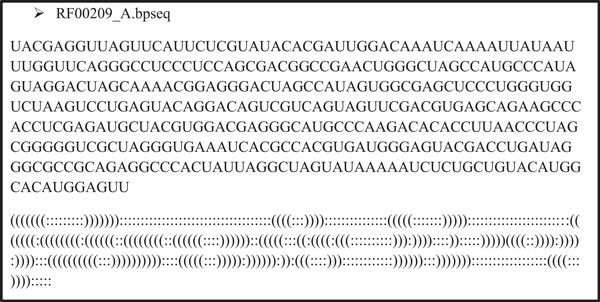
**RF00209_A sequence and secondary structure**. RF00209_A sequence and its experimental secondary structure from RFAM database. In the bracket view representation, bases that are hydrogen bonded with other bases are represented by a "(" or a ")" and a matching pair of "(" and ")" indicates that the bases at those positions are paired to be part of a secondary structure. Unpaired nucleotide bases are represented by a ":" (colon).

Both the whole and the assembled predicted structures are compared to the known structure to obtain their respective prediction accuracies so that we can assess to what degree the chunking method can preserve the prediction accuracy of the program when applied without any segmentation. Figure 11 shows the RF00209_A nucleotide sequence along with the bracket view of its experimentally known secondary structure. In the bracket view representation, bases that are hydrogen bonded with other bases are represented by a "(" or a ")", and a matching pair of "(" and ")" indicates that the bases at those positions are paired to be part of a secondary structure. Unpaired nucleotide bases are represented by a ":" (colon).

Various statistical tests are applied to the accuracy analysis for the different chunking methods including t-tests, Pearson correlation analysis, and the non-parametric Friedman tests. We use the statistical functions provided by MATLAB [22]. Metrics of interests include: (1) accuracy chunking (AC), which is the accuracy of the predicted structure assembled from the chunks when compared with the known secondary structure; (2) accuracy whole (AW), which is the accuracy of the predicted structure obtained from the whole sequence when compared with the known secondary structure; and (3) accuracy retention (AR), which is the ratio between AC and AW. While AC and AW reflect accuracies of the particular prediction in use with and without chunking, AR tells us how well a particular chunking method retains the accuracy of the original prediction program.

AC and AW are given by the percentage agreement of the predicted structure with the known real structure calculated as:

(4) $$100*\frac{\left[a+2*b\right]}{n}$$

where *a *and *b *represent respectively the number of unpaired bases and the number of base pairs in common between the two structures, and *n *is the length of the RNA sequence. Large AC and AW values (close to 100%) for a predicted structure mean that it is highly similar to the real structure.

The accuracy retention (AR) is defined as:

(5) $$AR=\frac{AC}{AW}$$

AR provides a comparison of the prediction accuracies with chunking versus without chunking. Intuitively, we expect that a good chunking method would cause only a minimal loss of prediction accuracy after cutting the sequence and would have AR values somewhat less than but close to 1. However, we will see in the result section that in many cases the AR values turn out to be greater than 1, meaning that secondary structure predicted using chunking is more similar to the real structure than it is the secondary structure predicted by using the whole sequence. Several standard statistical tests, including t-tests, Pearson correlation analysis, and the non-parametric Friedman tests [23], are applied to analyze the AR values for the different chunking methods.

### Adapting multiple searching paths to MapReduce

Given an RNA sequence, the search for the best set of chunking parameters (i.e., maximum chunk length *c*, chunking method, minimum stem length L, and maximum gap length G) requires us to traverse or search a multi-level tree (i.e. the chunking tree in Figure 3.b). In the chunking tree, each path from the root (RNA sequence) to the leaves (RNA chunks) represents a set of parameter values of the chunking method (i.e., *c*, L, and G). The overall workflow (including the chunking, prediction, reconstruction, and analysis steps) naturally adapts to fit into the MapReduce (MR) paradigm and can be easily implemented with Hadoop for which the chunking and predictions can be solved by multiple mappers while the reconstruction and the analysis are done by a single reducer. In our framework, each MR job is designed to partially traverse the multi-level tree. Multiple MR jobs can be executed in parallel to explore the whole tree. The multiple searching paths combine attributes of both breadth-first search (performed by multiple MR jobs in parallel) and depth-first search (performed by a single MR job). While traversing the tree with multiple MR jobs, we can explore the impact of different chunking methods as well as different *c*, *L *and *G *values for a given sequence. An example of an MR job is shown in the circled part of Figure 3.b, for which we assume the centered chunking method, with fixed *c *= 60 bases, and we vary *L *and *G *between 3 and 8 and between 0 and 8 respectively. As previously outlined, for a sequence and a combination of parameters, the mappers perform the chunking and predictions. The input to each mapper is a 〈*k*_1_*, v*_1_〉 value pair, in which *k*_1 _is the ID of the sequence, and *v*_1 _is the chunking parameters' values (including the chunking method). Each mapper cuts the sequence according to the chunking parameters values in the chunking step by identifying a variable number of chunks meeting the parameter requirements. Note that each combination of parameters (each branch of the tree) can result in a variable number of chunks. Each mapper performs the prediction on one or more chunks using a certain prediction program. Here we use five secondary structure prediction programs capable of predicting pseudoknots (IPknot [24], pknotsRG [25], HotKnots [3], NUPACK [26], and PKNOTS [8]) and two programs that do not include this capability (i.e., UNAFOLD [27] and RNAfold [28]). Other programs can be easily used in our framework as a plug-and-play software module. After the prediction, each mapper outputs the list of 〈*k*_2_*, v*_2_〉 pairs as the intermediate output to reduce. The *k*_2 _is the ID of the whole secondary structure to which the predicted chunk belongs and *v*_2 _is the predicted secondary structure of the chunk. After the Hadoop runtime system groups all the values associated with the same key and passes the 〈*k*_2_*, list*(*v*_2_)〉 to the reducer, the reducer reconstructs the whole secondary structure of the sequence using all the *v*_2 _(predicted chunk structures) associated with the same *k*_2_. If required, the reducer analyzes the results in terms of their accuracy. After the accuracy has been computed, the reducer outputs the final results as a *list*(*v*_3_), in which *v*_3 _is the AR for reconstructed structures.

#### Granularity of mappers

**Figure 12:**
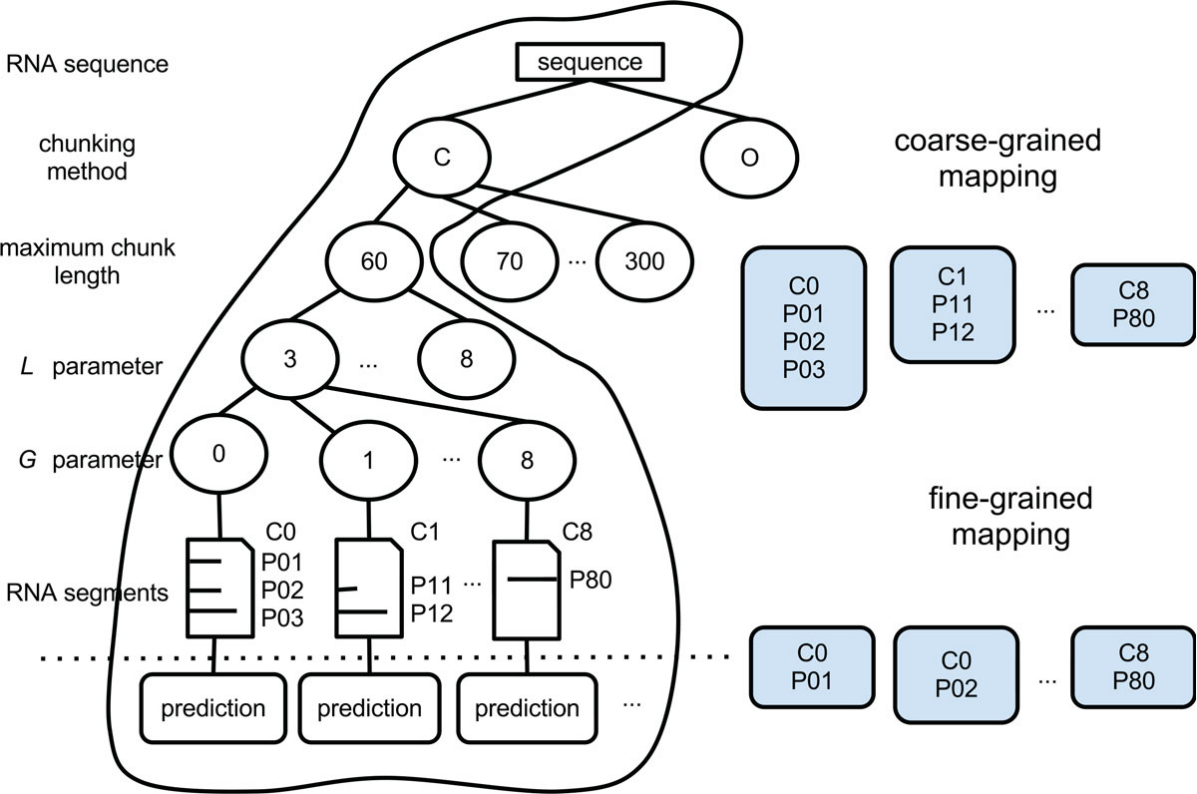
**Example of coarse-grained and fine-grained mapping**. Each mapper explores one branch of the tree and generates the set of segments as the output of the chunking program. The mapper predicts one or more segments generated based on the number of segments each mapper is assigned. Coarse-grained mappers explore one whole branch of the tree at the time. Fine-grained mappers predict one chunk at a time.

In general, a mapper is the process that runs on a processor which applies the map function to a specific key and value pair. In our framework, each mapper runs the chunking process on an RNA sequence with a given set of parameter values and then predicts one or multiple chunks. The granularity of the mapping can vary based on the number of chunks each mapper is assigned to predict. Our MR framework includes both a coarse-grained mapping and a fine-grained mapping as shown in Figure 12, in which each box represents a mapper. With the coarse-grained mapping, each mapper explores one branch of the chunking tree: it cuts the sequence into a set of segments based on a combination of *L *and *G *values and predicts all the segments it generates locally in order. With the fine-grained mapping, multiple mappers explore one branch of the chunking tree: each mapper cuts the same sequence into the same set of segments, but this time it predicts only one chunk that it generates. This means that if, for example, the sequence is cut into five segments, then there will be five mappers exploring the same branch of the chunking tree, replicating the chunking process but predicting only one distinguished segment of the five chunks available. The mappers determine which segment to predict based on a hash function; thus the mappers do not need to synchronize their work or directly agree on what chunk to predict. The hash function uses the ASCII value of the chunk identifier as the key and the identifier of each mapper as the value. The function selects the segments to mappers in a round robin fashion.

## Results and discussion

### Datasets and hardware platform

For the study of both accuracy and performance, we plug seven RNA secondary structure prediction programs into our framework for both the chunk-based predictions and the predictions of the same sequences without chunking (the whole sequence is taken). Five of the programs, IPknot [24], pknotsRG [25], HotKnots [3], NUPACK [26], PKNOTS [8] can predict both stem-loops and pseudoknots. The remaining two programs, UNAFOLD [27] and RNAfold [28], can predict stem-loops only. We consider both the centered (C) and optimized (O) chunking methods and compare them against the naïve regular method (R) as a reference. We also consider a wide range of parameter settings with maximum chunk length *c *from 60 to 150 bases, minimum stem length *L *from 3 to 8, and maximum gap length *G *from 0 to 8.

To study the framework accuracy, we use two datasets of sequences which have previously established secondary structures. The first dataset, compiled from the RFAM database, consists of 50 non-pseudoknotted sequences and the lengths of sequences range from 127 to 568 bases. The second dataset, compiled from the RFAM and Pseudobase++ [21,29] databases, consists of 23 pseudoknotted sequences, and the lengths of the sequences in this dataset range from 77 to 451 bases. Note that there are no large datasets of experimentally determined RNA secondary structures including pseudoknots, and to the best of our knowledge the one used in this paper is one of the few available to the public for free.

To study the framework performance, we use a smaller dataset of longer sequences (i.e., 14 RNA sequences from the virus family *Nodaviridae*) for which the secondary structures are not known. We assume pseudo-knots may be present and use the above-mentioned five prediction programs that are capable of capturing pseudoknots and we report only performance values but not accuracy. Because these RNA sequences are long (each has about 1300 to 3200 bases) and contain possible pseudoknots, none of the available programs can predict the secondary structures for the entire sequences. The use of the MapReduce framework is vital for the exhaustive, efficient exploration of the tree branches.

We ran the MapReduce framework on a cluster composed of 8 dual quad-core compute nodes (64 cores), each with two Intel Xeon 2.50 GHz quad-core processors. A front-end node is connected to the compute nodes and is used for compilation and job submissions. A high-speed DDR Infiniband interconnect for application and I/O traffic and a Gigabit Ethernet interconnect for management traffic connects the compute and front-end nodes. Our implementation is based on Hadoop 0.20.2.

### Accuracy

There are three main questions that we want to answer in regard to the effects of our chunk-based approaches on the accuracy of various established secondary structure prediction programs. First, we want to evaluate to what extent chunk-based predictions retain the prediction accuracy. Second, we want to identify whether the capability of a chunking method to retain the prediction accuracy might decline with increasing sequence lengths. Third, we want to assess the extent to which the inversion based chunking methods (C and O) outperform the naïve chunking method (R), and whether there is any difference in accuracy between the C and O chunking methods.

**Figure 13:**
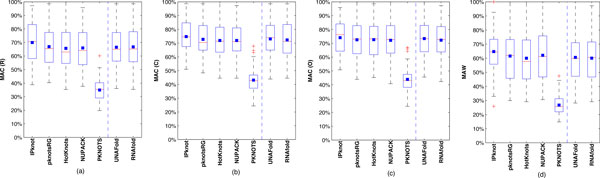
**MAC and MAW for chunking methods (R,C,O) for 50 sequences**. MAC and MAW values obtained using the prediction programs IPknot, pknotsRG, HotKnots, NUPACK, PKNOTS, RNAfold, and UNAFOLD for the dataset of 50 non-pseudoknotted sequences.

**Figure 14:**
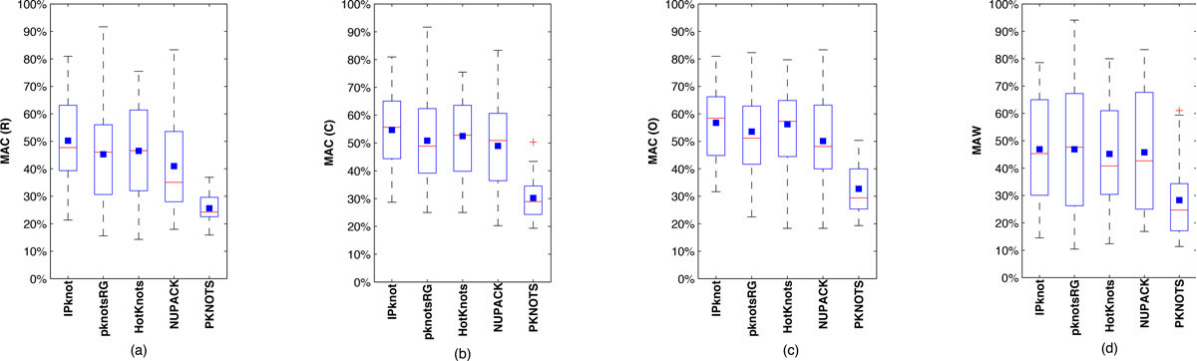
**MAC and MAW for chunking methods (R,C,O) for 23 sequences**. MAC and MAW values obtained using the prediction programs IPknot, pknotsRG, HotKnots, NUPACK, and PKNOTS for the dataset of 23 pseudoknotted sequences.

To assess how well the predictions based on chunking agree with known RNA structures, we measure the maximum AC (MAC) values of the sequences in the two datasets. Figures 13 and 14 present the box-and-whisker diagram for the two datasets and the three chunking methods - i.e., Figures 13.a,b, and 13.c show the box-and-whisker diagram for the regular, centered, and optimized methods respectively for the dataset of 50 non-pseudoknotted sequences. Figures 14.a,b, and 14.c show the box-and-whisker diagram for the regular, centered, and optimized methods respectively for the dataset of 23 pseudoknotted sequences. In the figures, the lower and upper quartiles are at the top and bottom boundaries of the box for the kernels; the median is the band inside the box; the mean is the black square; the whiskers extend to the most extreme data points or outliers; and outliers are plotted individually as "+" symbols.

As described in the Method section, the AC value for a predicted RNA structure is the percentage of agreement between the known structure and the structure obtained by concatenating the predicted structures of the chunks. Likewise, the AW value is the percentage of agreement between the known structure and the predicted structure when the whole sequence is used. These values indicate how closely the predicted structure resembles the real structure. A larger AC value means that the chunk-based predicted structure is more similar to the real structure. For a given dataset, prediction program, and chunking method, our MR framework collects multiple predicted structures associated with different *c*, *L*, and *G *parameters. The MAC value for a sequence is the maximum AC value, which gives the highest accuracy that can be attained for that sequence by the chunking method and the specific prediction program employed. In Figures 13.d and 14.d, the AW of the sequences in the two datasets are presented respectively. From these figures, it appears that most of the prediction methods have similar accuracy ranges regardless of the chunking method used and whether the prediction was obtained with the whole sequence or with the chunks; however, the PKNOTS program produces somewhat lower accuracies. This lower accuracy is quite expected because PKNOTS is actually the earliest algorithm allowing for pseudoknot prediction. The other prediction programs with pseudoknot prediction capability that have developed afterwards have incorporated improvements over the original PKNOTS.

From Figures 13 and 14, the prediction accuracies with chunking (MAC values in (a) - (c)) appear to be higher than those without (AW values in (d)), suggesting that the prediction accuracy, on average, can be enhanced by sequence segmentation. To get a clearer characterization of the effect of sequence segmentation, we carry out statistical tests on the maximum accuracy retention (MAR) obtained for each RNA sequence over the *c*, *L*, and *G *parameters. In the majority of the sequences in our dataset, the MAR turns out to be greater than 1. With a one-sample t-test, we test whether the mean MAR is significantly greater than 1 with p-value > 0.05. Tables 1 and 2 display the means, standard deviations, and p-values for the non-pseudoknotted and pseudoknotted sequences respectively.

**Table 1:** MAR statistics for 50 non-pseudoknotted sequences.

Cut	Regular			Centered			Optimized		
**Prediction**	**Mean**	**Stdev**	**p**	**Mean**	**Stdev**	**p**	**Mean**	**Stdev**	**p**

IPknot	1.13	0.32	0.002	1.23	0.36	0.000	1.21	0.36	0.000
pknotsRG	1.19	0.50	0.005	1.27	0.49	0.000	1.27	0.47	0.000
HotKnots	1.19	0.48	0.003	1.32	0.50	0.000	1.33	0.50	0.000
NUPACK	1.12	0.34	0.010	1.23	0.41	0.000	1.24	0.41	0.000
PKNOTS	1.33	0.19	0.000	1.65	0.35	0.000	1.70	0.35	0.000
UNAFold	1.19	0.49	0.003	1.31	0.47	0.000	1.31	0.46	0.000
RNAfold	1.19	0.46	0.002	1.31	0.48	0.000	1.30	0.45	0.000

Mean and standard deviations of MAR for regular, centered, and optimized chunking methods over 50 non-pseudoknotted sequences, and the corresponding p-values of the t-test for mean MAR *>*1.

**Table 2:** MAR statistics for 23 pseudoknotted sequences

Cut	Regular			Centered			Optimized		
**Prediction**	**Mean**	**Stdev**	**p**	**Mean**	**Stdev**	**p**	**Mean**	**Stdev**	**p**

IPknot	1.19	0.48	0.037	1.33	0.62	0.009	1.40	0.64	0.004
pknotsRG	1.21	0.84	0.116	1.39	0.99	0.036	1.48	1.00	0.016
HotKnots	1.11	0.41	0.098	1.32	0.71	0.021	1.43	0.80	0.009
NUPACK	0.93	0.18	0.955	1.14	0.39	0.071	1.17	0.35	0.032
PKNOTS	1.16	0.20	0.003	1.29	0.26	0.000	1.38	0.29	0.000

Mean and standard deviations of MAR for regular, centered, and optimized chunking methods over 23 pseudoknotted sequences, and the corresponding p-values of the t-test for mean MAR *>*1.

For non-pseudoknotted sequences, the mean MAR is significantly greater than 1 for all three chunking methods, whereas the mean MAR values for the pseudoknotted sequences are greater than 1 for the C and O chunking methods. With the R chunking method, one of the mean MAR values (with NUPACK) falls below 1 to 0.93. Looking at all the p-values, one can conclude that the average prediction accuracy attained with segmentation is not significantly less than that without. With the inversion based C and O chunking methods, we can conclude that the average prediction accuracies attained with segmentation are at least as good as, and often even better than, those without segmentation.

While the above results show that sequence segmentation will not reduce prediction accuracy on average, we still need to examine whether the MAR values would decline as the whole sequence length grows, because a declining trend would imply that the accuracy retention will deteriorate when the segmentation approaches are applied to longer RNA sequences. To this end, for each dataset, chunking method, and prediction program, we perform the Pearson correlation analysis on the MAR values of the sequences [30]. For each dataset, we report both the correlation coefficient r and corresponding p-value between MAR and sequence length. If the r value is close to -1, it means that MAR and sequence lengths are negatively correlated, implying a decline in accuracy retention of the chunking method. If the associated p value is less than 0.05, we consider the correlation statistically significant; otherwise the correlation is not significant.

**Figure 15:**
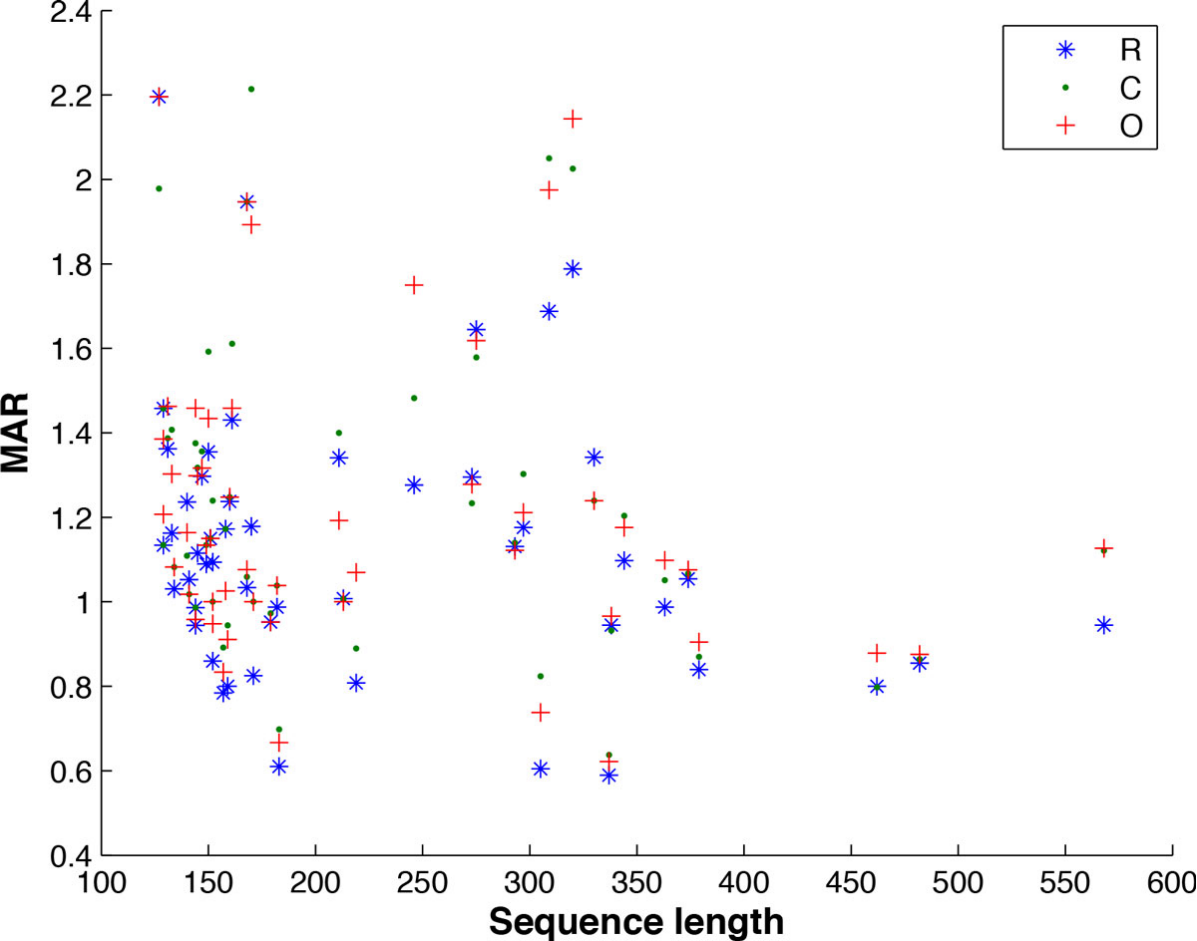
**MAR values for IPknot on non-pseudoknotted sequences**. Scatter plot of MAR values versus sequence lengths for the IPknot program. Similar scatter plots for the other prediction programs have been examined and no statistically significant negative correlation has been detected in any of these plots.

Figure 15 is a scatter plot of MAR values versus sequence lengths for one of the prediction programs, IPknot. Similar scatter plots for the other prediction programs have also been examined and no statistically significant negative correlation has been detected in any of these plots. Table 3 presents the correlation coefficients r and their corresponding p-values when the null hypothesis of no correlation is tested against the alternative hypothesis of having a negative correlation. These p-values indicate that no significant negative correlation has been detected for any of the prediction programs and chunking methods. We therefore do not expect any substantial decline in accuracy retention of our chunking methods while sequence length increases.

**Table 3:** MAR correlation coefficients (r) and p-values (p)

		Non-pseudoknotted sequences			Pseudoknotted sequences		
**Prediction**		**R**	**C**	**O**	**R**	**C**	**O**

IPknot	r	-0.2077	-0.2141	-0.1629	0.1379	0.1572	0.0841
	p	0.1478	0.1354	0.2582	0.5303	0.4738	0.7028
pknotsRG	r	-0.1550	-0.0670	-0.0756	0.2030	0.1971	0.1987
	p	0.2825	0.6437	0.6018	0.3528	0.3673	0.3634
HotKnots	r	-0.1434	-0.0476	-0.0732	-0.0360	-0.0669	-0.0855
	p	0.3204	0.7428	0.6136	0.8705	0.7618	0.6982
NUPACK	r	-0.1622	-0.0137	-0.0059	-0.0532	0.0666	-0.1331
	p	0.2604	0.9249	0.9676	0.8340	0.7928	0.5986
PKNOTS	r	0.4598	0.7053	0.7045	-0.0233	0.1001	0.0597
	p	0.0008	0.0000	0.0000	0.9294	0.7023	0.8199
UNAFold	r	-0.1449	-0.1055	-0.1311			
	p	0.3155	0.4658	0.3643			
RNAfold	r	-0.1056	-0.0646	-0.0538			
	p	0.4654	0.6559	0.7104			

Correlation coefficients (r) between MAR and sequence lengths and corresponding p-values (p) when testing for a negative correlation.

Given the three chunking methods considered (i.e., regular, centered, and optimized) we also want to determine which among them is better at retaining the accuracies of the various prediction programs. For this purpose, we examine each sequence in our two datasets and keep track of which chunking method produces the highest MAR. Table 4 gives the total counts of sequences attaining the highest MAR for each of the chunking methods. If more than one chunking method gets the same highest MAR for one sequence, we split the count of this sequence equally among the methods. We can see that the sequence counts in Table 4 for the centered and optimized (C and O) methods are higher than those for the regular method (R).

**Table 4:** Count and rank sum of sequences

	Non-pseudoknotted sequences						Pseudoknotted sequences					
**Prediction**	**R-co**	**R-rs**	**C-co**	**C-rs**	**O-co**	**O-rs**	**R-co**	**R-rs**	**C-co**	**C-rs**	**O-co**	**O-rs**

IPknot	7.7	74.0	24.7	115.5	17.7	110.5	2.0	32.0	7.0	47.0	14.0	59.0
pknotsRG	6.3	71.5	22.3	115.5	21.3	113.0	2.0	30.0	7.0	50.0	14.0	58.0
HotKnots	4.0	68.0	22.0	115.5	24.0	116.5	1.0	32.0	6.0	47.0	16.0	59.0
NUPACK	4.0	68.5	17.0	115.5	29.0	120.0	1.3	24.5	7.3	40.5	9.3	43.0
PKNOTS	1.0	55.5	17.5	114.5	31.5	130.0	1.5	21.0	4.0	35.5	11.5	45.5
UNAFold	4.0	67.0	25.0	118.0	21.0	115.0						
RNAfold	4.0	65.0	18.0	112.5	28.0	122.5						

Count (co) and rank sum (rs) of sequences attaining the highest MAR with each chunking method for the various prediction programs.

To see if there are any differences among the accuracy retention capabilities among the three cutting methods, we perform the Friedman test for each dataset and each prediction program. The Friedman test is a non-parametric statistical test based on rank sums [23] and requires ranking the MAR attained by each chunking method for each prediction program and each sequence in our data sets. The method producing the lowest MAR is given a rank of 1 and the method producing the highest MAR is given a rank of 3. Again, the ranks are averaged for ties. Table 5 shows the p-values of the Friedman tests in the "R-C-O" columns. From these very low p-values, we can conclude that there are significant differences among the three methods.

**Table 5:** P-values from the Friedman test

	Non-pseudoknotted sequences				Pseudoknotted sequences			
**Prediction**	**R-C-O**	**C-O**	**R-C**	**R-O**	**R-C-O**	**C-O**	**R-C**	**R-O**

IPknot	9.21E-06	5.27E-01	2.43E-05	4.02E-04	2.16E-05	4.55E-02	4.50E-03	3.74E-05
pknotsRG	1.82E-07	5.27E-01	1.86E-06	9.72E-06	3.65E-06	1.84E-02	1.08E-04	9.62E-05
HotKnots	8.80E-09	6.47E-01	4.20E-07	5.36E-07	2.47E-05	1.84E-02	1.30E-03	1.62E-04
NUPACK	5.16E-09	2.17E-01	5.79E-08	1.41E-06	3.70E-04	5.64E-01	9.11E-04	1.30E-03
PKNOTS	1.22E-15	2.69E-02	3.56E-10	1.18E-11	6.78E-06	6.70E-03	5.32E-04	1.83E-04
UNAFold	5.25E-09	2.74E-01	6.91E-07	2.96E-07				
RNAfold	1.18E-08	8.82E-01	1.29E-06	1.81E-07				

P-values from the Friedman test to compare the accuracy retention of the three chunking methods as well as the posthoc pairwise comparison tests.

Because the Friedman test does not reveal whether any one method is significantly better than another, we also perform the post-hoc pairwise comparison test on each pair of the three chunking methods in order to confirm that the inversion based centered and optimized chunking methods are indeed superior to the naïve regular method. The p-values, shown in the "R-C," "R-O," and "C-O" columns, indicate that both the centered and optimized methods are better than the regular method. Furthermore, there are no significant differences between the centered and optimized chunking methods except when PKNOTS is applied to the pseudoknotted sequences.

The results above demonstrate that, for a variety of secondary structure prediction programs, our segmentation approach for handling the long RNA sequences can retain and even enhance the average prediction accuracy. Furthermore, using the inversion based C and O methods to cut the sequence will produce better prediction accuracy than the naïve R method. More questions remain to be answered and are part of our current research.

Our current investigations focus on the following two questions. First, we want to study how we should choose the parameters *c*, *L*, and *G *to maximize the accuracy retention. We have been conducting studies to identify how the prediction accuracy correlates with these parameters. Some of the results have been reported in preliminary work of the group [12,13]. So far we have not found any definitive criteria that work for all sequences in general. Rather, the nucleotide base composition and length of the individual sequence, as well as the sequence length limitations imposed by the particular prediction program, need to be taken into account. Second, the fact that segmentation can in many cases improve the prediction accuracy for an RNA sequence is somewhat counter-intuitive. One possible explanation is that secondary structure prediction algorithms are generally based on global minimal free energy, resulting in the most thermodynamically stable isoforms. However, these structures may not be most favorable for biological functions, which often require RNAs to interact with other molecules or unfold during replication. Our results suggest that local structures formed by pairings of bases in close proximity, rather than the global energies, may better correlate with the real structures of large RNA molecules. This hypothesis is being tested in coauthor Johnson's molecular virology lab using the virus family *Nodaviridae*.

**Figure 16:**
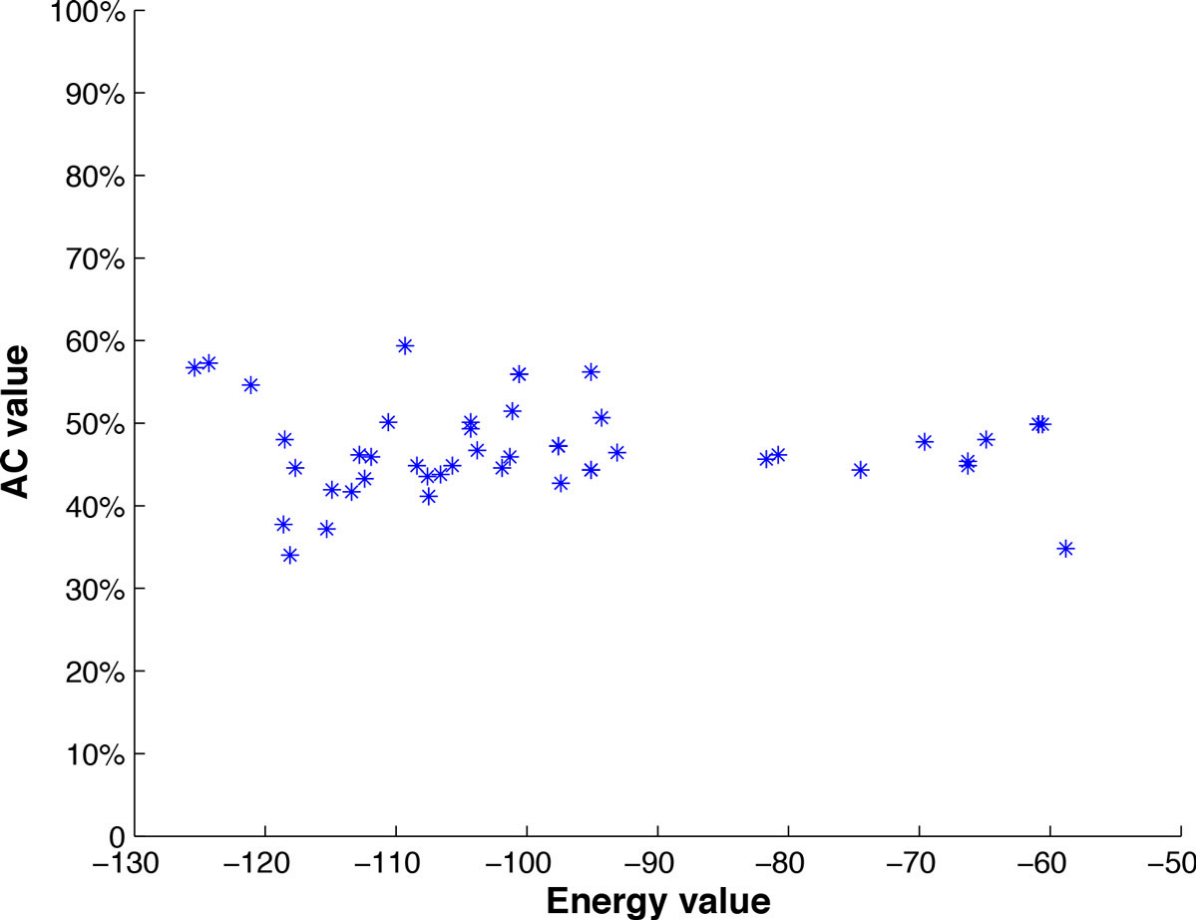
**Accuracy versus free energy for the RFAM sequence RF0002_A**. Scatter plot of prediction accuracy versus free energy of different predicted structures of the RFAM sequence RF0002_A using the centered and optimized chunking methods with different *L *and *G *parameters with maximum chunk length of 100. Correlation coefficient *r *= 0.11.

The above idea also prompted us to initiate a study on the correlation between accuracy and the free energy of our chunk-based predicted structures. Since there are no straightforward mechanisms within the current prediction programs to compute the free energy of a given structure other than those outputted by the program, we try to obtain the overall free energy of our chunk-based predictions by simply summing the free energies associated with the chunks. Among several examples that we have studied to date, most do not show any statistically significant relationship to support the idea that global structures with lower free energies are more similar to the known structure. One example scatter plot of the prediction accuracy versus free energy of different predicted structures of the sequence RF000_2A using the centered and optimized chunking methods with different L and G parameters with maximum chunk length of 100 is shown in Figure 16. The correlation coefficient is found to be positive 0.11, which is against the expectation of a negative correlation. We anticipate that this line of investigation will require more coordinated efforts with the developers of the various prediction programs to establish appropriate ways of computing the free energies of any given predicted-or experimentally-determined structure.

### Performance

For the performance analysis, we use a smaller dataset of longer sequences from the virus family *Nodaviridae *[31,32] and we explore a wider range of parameter values. The virus family *Nodaviridae *is divided into two genera: *alphanodaviruses *that primarily infect insects and *betanodaviruses *that infect only fish. These viruses share a common genome organization, namely a bipartite positive strand RNA genome (i.e., mRNA sense). The longer genome segment RNA1 (ranging in size from 3011 to 3204 nucleotide bases) encodes the RNA-dependent RNA polymerase that catalyzes replication of both genome segments, while the shorter RNA 2 (ranging in size from 1305 to 1433 nucleotide bases) encodes the precursor of the viral capsid protein that encapsidates the RNA genome. The 14 sequences we analyze in this paper are identified as follows: *Boolarra virus *(BoV) RNA2 (1305 nucleotide bases), *Pariacoto virus *(PaV) RNA2 (1311), *Nodamura virus *(NoV) RNA2 (1336), *Black beetle virus *(BBV) RNA2 (1393), *Flock House virus *(FHV) RNA2 (1400), *Striped jack nervous necrosis virus *(SJNNV) RNA2 (1421), *Epinephelus tauvina nervous necrosis virus *(ETNNV) RNA2 (1433), BoV RNA1 (3096), PaV RNA1 (3011), BBV RNA1 (3099), ETNNV RNA1 (3103), FHV RNA1 (3107), SJNNV RNA1 (3107), NoV RNA1 (3204). These sequences are sorted based on their increasing lengths, and this order is preserved in all the figures and tables presented below. There are three important questions that we want to answer when measuring performance. First, we want to quantify the time spent for exploring the several branches of the search trees for these 14 sequences using each of the two chunking methods (centered or optimized) and for the granularity of the mapping (coarse-or fine-grained). Second, we want to identify how the time is spent for each search in terms of map, reduce, and data shuffling among processors. Third, we want to measure the efficiency of the search and look for those aspects of the search that can impact performance.

We measure the total time needed to explore the chunking tree of each sequence using either the centered or optimized methods and with either coarse-grained or fine-grained mapping. The total time includes the time needed for chunking and prediction (map time), reconstruction (reduce time), exchange of predictions among nodes (shuffling time), and any overhead due to load imbalance and synchronizations. Note that the total time does not include the time needed for analysis since the secondary structures of the sequences considered here are not known experimentally; thus an analysis in terms of accuracy is not feasible. We use IPknot for our predictions since it is the most recently implemented program and its accuracy values are very high in the previous section.

**Figure 17:**
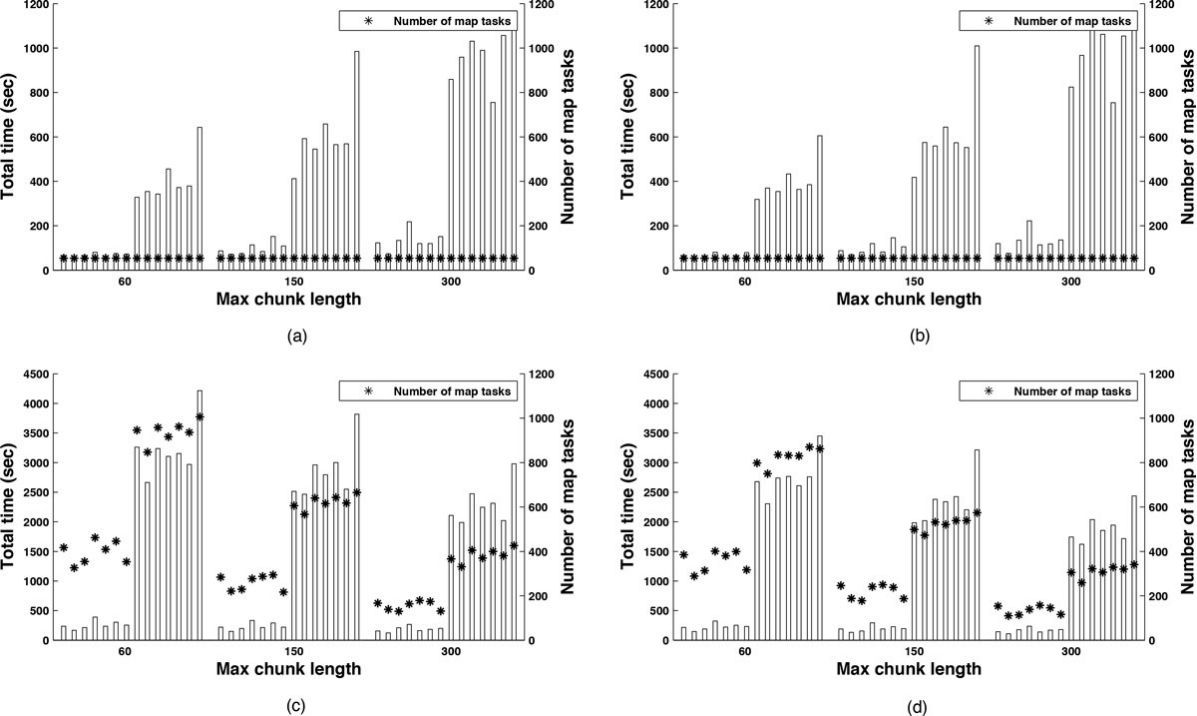
**Total MapReduce times for different methods and mapping**. Total time in seconds for coarse- vs. fine-grained mapping and centered vs. optimized methods, i.e., (a) Coarse-grained mapping using centered method; (b) Coarse-grained mapping using optimized method; (c) Fine-grained mapping using centered method; and (d) Fine-grained mapping using optimized method.

Each of the four subfigures in Figure 17 shows the total times in seconds for exploring the prediction trees (left y-axes) and the number of map tasks (right y-axes) for the 14 sequences when a maximum chunk length of 60, 150, and 300 bases is used. In each subfigure there are three groups of times, one for each maximum chunk length. Each group lists the 14 sequences sorted based on their length in nucleotide bases. More specifically, Figure 17.a presents the times and number of map tasks when the coarse-grained MR implementation and the centered method are used; Figure 17.b presents the times and number of map tasks when the coarse-grained MR implementation and the optimized method are used; Figure 17.c presents the times and number of map tasks when the fine-grained MR implementation and the centered method are used; and Figure 17.d presents the times and number of map tasks when the fine-grained MR implementation and the optimized method are used. As already presented above, when using coarse-grained mapping, each mapper performs the chunking for the assigned sequence using a set of parameter values for the max length of stems (L) and gap sizes (G). The mapper then predicts the secondary structures of all its local chunks. This results in the exploration of a whole branch of the tree by the mapper. The total number of branches (and map tasks) is given by the combinations of *L *and *G *values (i.e., 54). When using fine-grained mapping, chunking of a sequence based on a set of *L *and *G *values is performed across mappers and mappers are assigned resulting chunks in a round-robin fashion. Computationally this is performed by replicating the chunking processes across mappers and by using a hash function to assign different chunks to different mappers. The number of chunks equals the number of map tasks and depends on the number of inversions identified in the chunking process.

When comparing centered vs. optimized chunking methods for the coarse-grained mapping, we observe that the two methods result in similar execution times (Figure 17.a and Figure 17.b). Table 6 quantifies the similarity for both subgroups (i.e. RNA2 and RNA1) which is within 3%. This observation is different from the previous work in which the centered method resulted in shorter execution times due to the fact that a different implementation of the chunking methods and a different program were used.

**Table 6:** Total times for RNA2 and RNA1 with coarse-grained mapping

Mean Total Time (sec)	RNA2(*∼*1300 bases)			RNA1(*∼*3100 bases)		
	**60**	**150**	**300**	**60**	**150**	**300**

Centered	68.7	99.0	134.3	410.7	618.0	971.7
Optimized	66.7	98.9	131.6	404.3	618.9	986.1
Opti./Cent.	0.97	1.00	0.98	0.98	1.00	1.01

Average total times for the seven sequences in RNA2 and in RNA1 for coarse-grained mapping using centered and optimized methods.

When comparing centered vs. optimized chunking methods for the fine-grained mapping, the optimized method results in a slightly lower execution time. As shown in Table 7, the execution times of fine-grained mapping when using the optimized chunking method for both subgroups (RNA2 and RNA1) is 11% to 18% slower than using the centered method. Table 8 shows the average number of chunks (i.e., map tasks) for both subgroups using centered and optimized methods. We can see that the optimized method results in 10% to 19% less chunks. The optimized method tends to cut sequences into fewer chunks, which leads to fewer map tasks and shorter MapReduce total times. This observation is different from the previous work in which the centered method results in shorter execution times due to the same reason we mentioned above [17].

**Table 7:** Total times for RNA2 and RNA1 with fine-grained mapping

Mean Total Time (sec)	RNA2(*∼*1300 bases)			RNA1(*∼*3100 bases)		
	**60**	**150**	**300**	**60**	**150**	**300**

Centered	257.7	232.3	185.7	3228.7	2871.7	2303.9
Optimized	226.6	197.4	164.6	2758.0	2366.0	1907.7
Opti./Cent.	0.88	0.85	0.89	0.85	0.82	0.83

Average total times for the seven sequences in RNA2 and in RNA1 for fine-grained mapping using centered and optimized methods.

**Table 8:** Average number of chunks for RNA2 and RNA1 with fine-grained mapping

Mean Total Time (sec)	RNA2(*∼*1300 bases)			RNA1(*∼*3100 bases)		
	**60**	**150**	**300**	**60**	**150**	**300**

Centered	395	258	155	939	622	383
Optimized	355	218	134	825	525	312
Opti./Cent.	0.90	0.84	0.86	0.88	0.84	0.81

Average number of chunks (i.e. number of map tasks) for the seven sequences in RNA2 and in RNA1 for fine-grained mapping using centered and optimized methods.

When comparing coarse-grained mapping vs. fine-grained mapping, we observe that coarse-grained mapping results in shorter execution time compared to fine-grained mapping, independent of the chunking method used. Also we observe the trend that when the maximum chunk length grows from 60 to 300, the time gain of coarse-grained mapping over fine-grained mapping decreases. The speedup of coarse-grained mapping over fine-grained mapping using the centered chunking method for RNA2 subgroup of sequences decreases from 3.75 to 1.38, and for RNA1 it decreases from 7.86 to 2.37. A similar behavior is observed for the optimized chunking method: speedup of coarse-grained mapping over fine-grained mapping for RNA2 subgroup of sequences decreases from 3.4 to 1.25, and for RNA1 it decreases from 6.82 to 1.93.

**Figure 18:**
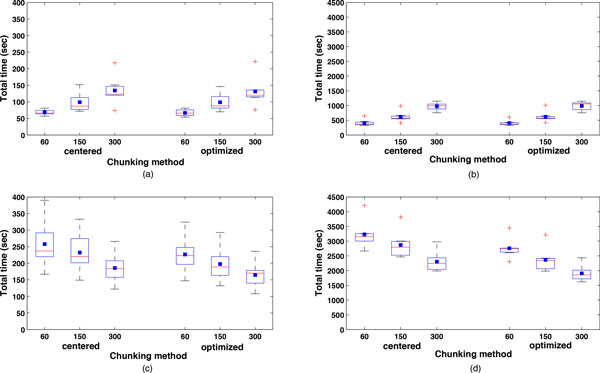
**Box-and-whisker diagram of total MR times**. Box-and-whisker diagram of total MR times for each subgroup of *Nodaviridae *sequences (RNA2 and RNA1) using centered and optimized methods with maximum chunk length of 60, 150, and 300, i.e., (a) Coarse-grained mapping on RNA2 (short sequences); (b) Coarse-grained mapping on RNA1 (long sequences); and (c) Fine-grained mapping on RNA2 (short sequences); and (d) Fine-grained mapping on RNA1 (long sequences).

The same trend of the total times is summarized in the box-and-whisker diagram of minimum, median, mean, and maximum execution time for each subgroup of sequences (RNA2 and RNA1) in Figure 18. More specifically, in Figure 18.a, we show the box-and-whisker diagram of the total times for the RNA2 subgroup of sequences (∼1300 nucleotides bases) using coarse-grained mapping, both centered and optimized methods, and maximum chunk lengths of 60,150, and 300. In Figure 18.b, we show a similar box-and-whisker diagram but for the RNA1 subgroup of sequences (∼3100 bases). In Figure 18.c, we show the box-and-whisker diagram of the minimum, mean, and maximum execution time for the RNA2 subgroup of sequences using fine-grained mapping, centered and optimized methods, and max chunk lengths of 60, 150, and 300. In Figure 18.d, we show a similar box-and-whisker diagram but for the RNA1 subgroup of sequences. We observe that for coarse-grained mapping, when using the centered and optimized methods, the average total times increase with the maximum chunk length at the rate of 2.0 for RNA2 and 2.4 for RNA1. On the contrary for the fine-grained mapping, when using centered and optimized methods, the average total times decrease with the maximum chunk length at the rate of 0.7 for both subgroup of sequences. This suggests that potentially for larger maximum chunk length and sequence lengths the fine-grained mapping can outperform the coarse-grained mapping in terms of performance.

**Figure 19:**
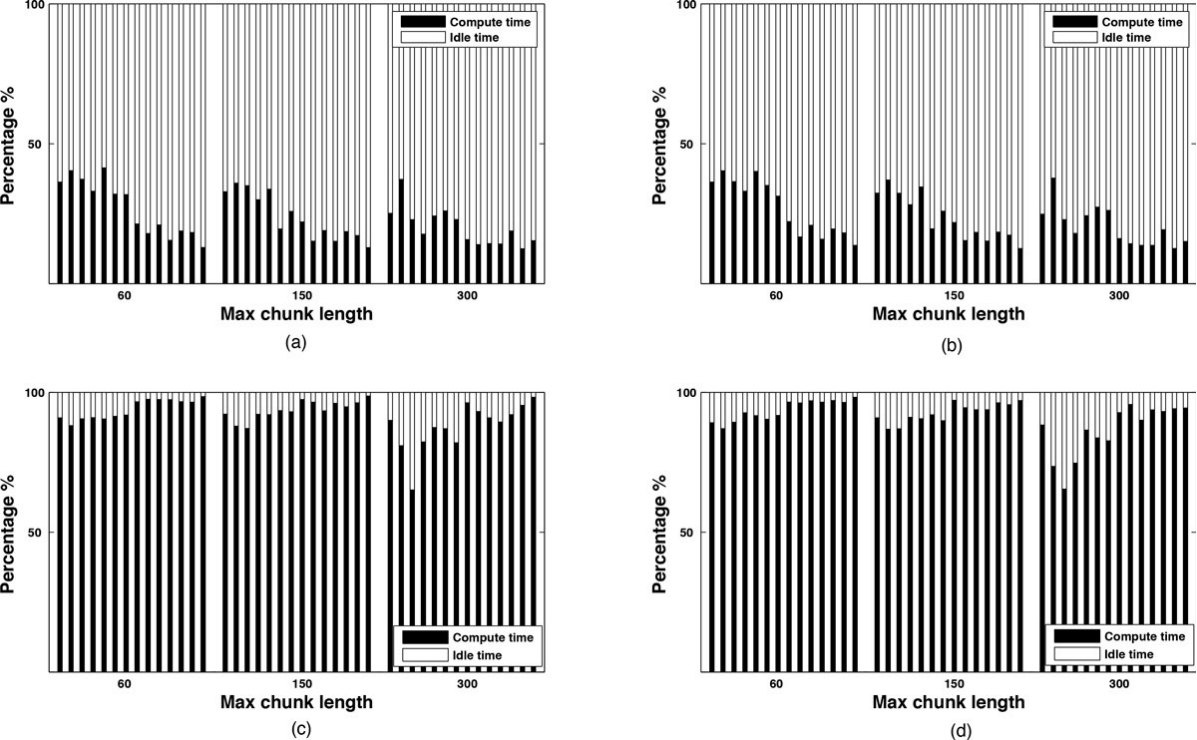
**Efficiency of map functions for different methods and mapping**. Percentages of compute and idle time in map function for coarse- vs. fine-grained mapping and centered vs. optimized methods, i.e., (a) Coarse-grained mapping using centered method; (b) Coarse-grained mapping using optimized method; (c) Fine-grained mapping using centered method; and (d) Fine-grained mapping using optimized method.

When decoupling the total time in its components, we observe that the time components for the reduce function and shuffling are very marginal compared to the times used for the mapping functions (around 1% of the total time). We also observe that, as we explore a prediction tree, some mappers are performing more work than others, resulting in idle time and low efficiency. The load imbalance among mappers depends on the granularity and chunking methods used. To better understand the causes of load imbalance we cut down the mapping times into compute time (i.e., chunking and predictions) and idle time (i.e., waiting for all the mappers to complete their predictions). Figure 19 shows the percentage of compute and idle times in map function for coarse-grained mapping vs. fine-grained mapping as well as for centered vs. optimized methods. More specifically, Figures 19.a and 19.b show the percentages for compute and idle times for the coarse-grained framework with the centered and optimized methods respectively; Figures 19.c and 19.d show the same percentages but for the fine-grained framework and the two chunking methods.

Independent of the maximum chunk length, Figure 19 shows how fine-grained mapping reaches better efficiency compared to coarse-grained mapping. In other words, with fine-grained mapping, the mappers spend more time doing real chunking and predictions. We observe in Figure 17 (left y-axes) how fine-grained mapping has a larger number of map tasks and each map task is shorter (it predicts only one chunk) making easier for the Hadoop scheduler to allocate the several tasks efficiently by using a first-in-first-out (FIFO) policy. On the other hand, coarse-grained mapping has a smaller number of map tasks and each map task is longer (all the sequence chunks of a given *L *and *G *combination are predicted by a single mapper). In this case, once the scheduler assigns a longer task to a mapper, it has to wait for its completion, even if the other mappers have generated their chunk predictions, before proceeding to the reduce phase. We also observe that as the maximum chunk length increases from 60 to 300 bases, the map efficiency tends to drop. More specifically, the average map efficiency for coarse-grained mapping decreases from 36% to 25% on RNA2 and from 18% to 15% on RNA1 when using centered or optimized chunking methods. The average map efficiency for fine-grained mapping decreases from 91% to 79% on RNA2 and from 97% to 93% on RNA1. This is due to the fact that the centered and optimized chunking methods tend to produce more chunks with shorter chunk lengths when using a maximum chunk length of 60. On the other hand, when using a maximum chunk 300, the same methods tend to produce fewer chunks each with longer lengths.

**Figure 20:**
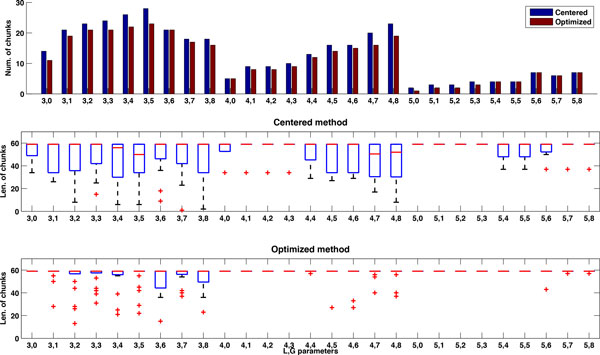
**Profile of *Nodamura virus* (NoV) RNA2 with maximum chunk length 60**. Number of chunks and chunk lengths for the *Nodamura virus *(NoV) RNA2 with centered and optimized methods and maximum chunk length of 60 bases.

**Figure 21:**
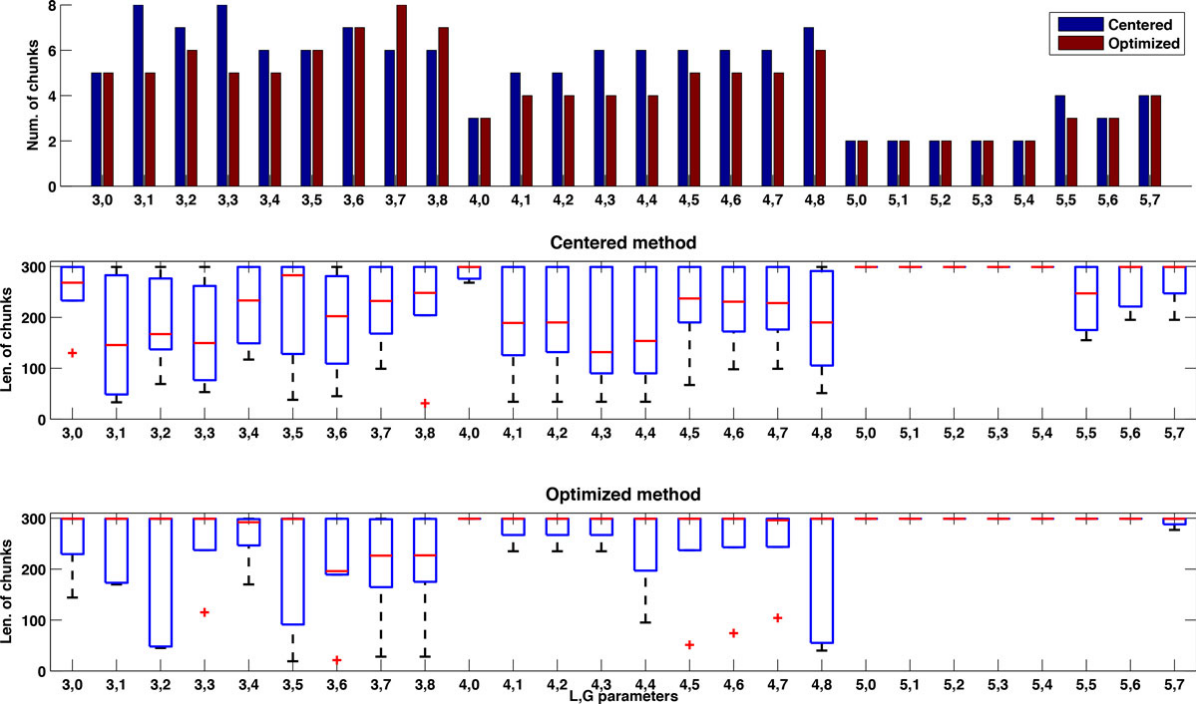
**Profile of Nodamura virus (NoV) RNA2 with maximum chunk length 300**. Number of chunks and chunk length for the *Nodamura virus *(NoV) RNA2 with centered and optimized methods and maximum chunk length of 300 bases.

Diverse chunk lengths within a prediction can also cause inefficiency. To study this phenomenon, we consider the *Nodamura virus *(NoV) RNA2 sequence which shows the largest drop in efficiency when moving from 60 to 300 max chunk lengths, as shown in Figure 19. Figures 20 and 21 show the number of chunks and their lengths (i.e., max, min and median) for the different *L *and *G *parameter combinations with centered and optimized methods when the maximum chunk length is equal to 60 (Figure 20) and when the length is equal to 300 (Figure 21). When the maximum length grows from 60 to 300, the number of resulting chunks for each combination of *L *and *G *parameters decreases. At the same time the length of each set of chunks increases as well as the length variability within the set of chunks for a defined combination of *L *and *G *values. Note that for some combinations of *L *and *G*, the chunking process does not identify any set of chunks and we do not report any result for these cases. This confirms our observation that as the number of chunks decreases, the chunk lengths increase but not homogeneously within a prediction, causing load imbalance and loss in efficiency. Selecting the shorter maximum length for the sake of efficiency is not always a wise decision: a maximum chunk length of 60 bases may be too short for the type of RNA sequences we are considering. In Figure 20, the median is very close to the maximum length of 60 for the centered methods, indicating that we are cutting out valuable parts of the inversion and ultimately of the secondary structures we are predicting.

The overall results suggest that the best set of parameter values to achieve higher accuracy, performance, and efficiency depend on multiple aspects including the input sequence and the available resources. Driven by these two aspects, in future work we will integrate an automatic selection of these values into our MR framework.

## Conclusions

In this paper, we propose a MapReduce-based, modularized framework that allows scientists to systematically and efficiently explore the parametric space associated with chunk-based secondary structure predictions of long RNA sequences. By using our framework we can observe how sequence segmentation strategies, directed by inversion distributions enable us to predict the secondary structures of large RNA molecules. Furthermore, the chunk-based predictions can, on average, attain accuracies even higher than those obtained from predictions using the whole sequence. The observations in this study have led to our hypothesis that local structures formed by pairings of bases in close proximity, rather than the global free energies, may better correlate with the real structures of large RNA molecules. This hypothesis will be tested by further computational and experimental investigations.

## Competing interests

The authors declare that they have no competing interests.

## Authors' contributions

BZ adapted and implemented the chunk-based RNA prediction process into MapReduce Hadoop, carried out the accuracy and performance experiments, and participated in drafting the manuscript. DY participated in the design, implementation and modification of the sequence segmentation strategies, conducted the statistical analyses of their accuracy retention, and helped to draft the manuscript. KLJ provided the nodavirus genome sequence data and their biological significance. MYL supervised the accuracy analyses and summarized the statistical results. MT oversaw the MapReduce implementation and performance analysis, and coordinated the organization and writing of the manuscript. All authors reviewed and participated in finalizing the manuscript.

## Declarations

The publication costs for this article were funded by the corresponding authors.

This article has been published as part of *BMC Structural Biology *Volume 13 Supplement 1, 2013: Selected articles from the Computational Structural Bioinformatics Workshop 2012. The full contents of the supplement are available online at http://www.biomedcentral.com/bmcstructbiol/supplements/13/S1.
